# Nursing Home, Ward and Worker Level Determinants of Perceived Quantitative Work Demands: A Multi-Level Cross-Sectional Analysis in Eldercare

**DOI:** 10.1093/annweh/wxac039

**Published:** 2022-06-23

**Authors:** Matthew L Stevens, Kristina Karstad, Leticia Bergamin Januario, Svend Erik Mathiassen, Reiner Rugulies, David M Hallman, Andreas Holtermann

**Affiliations:** Department of Musculoskeletal Disorders and Physical Workload, The National Research Centre for the Working Environment, Lersø Parkallé 105, 2100 København ØDenmark; Department of Musculoskeletal Disorders and Physical Workload, The National Research Centre for the Working Environment, Lersø Parkallé 105, 2100 København ØDenmark; Department of Occupational Health Sciences and Psychology, Centre for Musculoskeletal Research, University of Gävle, Kungsbäcksvägen 47, 801 76 Gävle, Sweden; Department of Occupational Health Sciences and Psychology, Centre for Musculoskeletal Research, University of Gävle, Kungsbäcksvägen 47, 801 76 Gävle, Sweden; Department of Musculoskeletal Disorders and Physical Workload, The National Research Centre for the Working Environment, Lersø Parkallé 105, 2100 København ØDenmark; Department of Public Health, University of Copenhagen, Øster Farimagsgade 5, 1353 København K, Denmark; Department of Occupational Health Sciences and Psychology, Centre for Musculoskeletal Research, University of Gävle, Kungsbäcksvägen 47, 801 76 Gävle, Sweden; Department of Musculoskeletal Disorders and Physical Workload, The National Research Centre for the Working Environment, Lersø Parkallé 105, 2100 København ØDenmark; Department of Sports Science and Clinical Biomechanics, University of Southern Denmark, Campusvej 55, 5230 Odense, Denmark

**Keywords:** eldercare workers, quantitative work demands

## Abstract

**Introduction:**

Perceived quantitative demands at work have been associated with poor mental and physical health, long-term sickness absence and subsequent early retirement. Identifying modifiable determinants of perceived quantitative demands at different levels of the organization is key to developing effective interventions. The aim of the study was to identify determinants of perceived quantitative demands at work and examine the extent to which they occur at different levels of the eldercare organisation (i.e. the worker, ward and nursing home levels).

**Methods:**

We collected data on 383 eldercare workers in 95 wards at 20 nursing homes in Denmark using workplace observations and questionnaires to workers and their managers. Perceived quantitative work demands were assessed using two items from the Copenhagen Psychosocial Questionnaire, II. We identified contributions to overall variability from the three organisational levels using variance components analysis, and examined associations between determinants at these three levels and quantitative demands.

**Results:**

Almost all (90.9%) the variability in perceived quantitative demands occurred between eldercare workers (within wards). Determinants significantly associated with lower quantitative demands were: having a job as a care helper, working fixed evening shifts, being born outside Denmark, having lower influence at work, higher quality of leadership and lower emotional demands. None of the investigated physical factors (e.g. resident handlings, push/pull tasks, step-count) were associated with perceived quantitative demands.

**Conclusion:**

We found that the variability in perceived quantitative demands occurred primarily between eldercare workers within wards. Our study indicates that psychosocial work environment factors are the strongest modifiable determinants of perceived quantitative demands in eldercare, while organisational factors related to job position, shift, and resident-staff ratio also play a role. Interventions should test if changes in these determinants can reduce perceived quantitative demands at work in eldercare.

**What’s important about this paper?**

The perceived amount of work required of a worker relative to the time available to conduct the work, the quantitative work demand, is a strong predictor for poor health and sickness absence, but the role of organizational level factors in this association is not well understood. This study found that, in eldercare organizations, most of the variability in perceived quantitative demands occurred primarily between eldercare workers within wards. Psychosocial work environment factors were the strongest modifiable factors associated with perceived quantitative demands, while organisational factors related to job position, shift, and resident-staff ratio also play a role. These findings provide insight into which factors at the different levels of the organizations we should intervene on when aiming for reducing perceived quantitative demands among eldercare workers.

## Introduction

Eldercare is an important profession, with quality long-term care services being one of the core principles of the European Pillar for Social Rights (European Commission, 2017). Moreover, with the proportion of Europeans aged 80+ expected to more than double by 2070 (European Commission *et al.*, 2018), the demand for eldercare will increase. This increased demand for eldercare is being accompanied by rising retirement ages, requiring eldercare workers to be able to work to older ages (European Commission, 2012). These factors together add strain to an occupational sector that is already dealing with a less attractive image due to general perceptions of poor working conditions, low socioeconomic status, low wages and high staff turnover (Bishop *et al.*, 2008; EU Skills Panorama, 2014). To strengthen the viability of the sector, research-based initiatives for improving the working conditions of eldercare workers are key (European Commission, 2012; Clausen *et al.*, 2014b; Roen *et al.*, 2018; European Commission *et al.*, 2018).

An important factor in eldercare work is perceived quantitative work demands (Gao *et al.*, 2014; Clausen *et al.*, 2014b) – referring to the perceived amount of work required of a worker relative to the time available to conduct the work (Kristensen *et al.*, 2004). In Denmark, eldercare workers are in the top 10 list (out of 72 jobs) scoring highest on questions related to quantitative demands (Arbejdstilsynet, 2022) and there has been significant debate around the increasing quantitative demands for eldercare workers due to increasing number of elderly in need of care, concerns that the ratio between eldercare workers per eldercare residents is too low, and an expected shortage of eldercare workers in the coming years.

Quantitative demands, as evaluated with self-administered questionnaires, describe how much a person is expected to achieve in his/her work tasks, measuring the discrepancies between the tasks’ requirements and the time given to perform such tasks in an adequate way (Burr *et al.*, 2019). Numerous studies have linked high quantitative demands with a range of different health outcomes such as poor mental and physical health, long term sickness absence and subsequent early retirement (Schütte *et al.*, 2014; Slany *et al.*, 2014; Freimann *et al.*, 2016). However, there do seem to be protective factors that nullify this association. For example, several studies have only identified this association in the presence of low job control (Rugulies *et al.*, 2010; Madsen *et al.*, 2017). Quantitative demands cannot be completely distinguished from other closely related concepts such as work pace or workability, and we consider this a strength of the concept quantitative demands. Perceived quantitative demands is inherently a balance between the’actual’ work demands on one side and the person’s resources and ability to accomplish those demands on the other. The ‘balance’ (or lack of balance) between the ‘actual’ work demands and the resources and ability of the worker is likely important for risk of impaired health.

To develop interventions for quantitative demands in eldercare work, we need a better understanding of the determinants of quantitative demands. Determinants in a workplace setting may arise from either the individual workers (individual determinants) or from the work/workplace (organisational determinants). Furthermore, determinants that arise from the work/workplace may also occur at various organisational levels within the workplace (Sallis *et al.*, 2006). For instance, in eldercare, three nested levels may be identified in the organizational hierarchy. These levels are nursing homes, wards (within nursing homes), and workers (within wards), and differences in determinants may occur at all three levels. An example of a worker level determinant is work tasks, which often vary between workers. However, some tasks may be common to all workers on a specific ward, perhaps as a nature of the residents in the ward (i.e. residents of a dementia ward will likely have different care requirements compared to a somatic ward), causing work tasks to differ according to the wards in which the workers are employed. These differences will therefore occur at the ward level. As such, to fully understand the determinants of quantitative demands and how best to modify these determinants, we must also understand at which organisational level these determinants occur and how they impact upon quantitative demands.

Although the importance of understanding determinants across all levels of an organisation is widely acknowledged (Sallis *et al.*, 2006), most research in eldercare has not considered the role of organisational level. As such, the knowledge about determinants of quantitative demands at different levels of the organization is very limited (Hansen *et al.*, 2015). Thus, the aim of the study was to identify determinants of perceived quantitative demands at work and determine the extent to which they occur at different levels of the eldercare organisation (i.e. the worker, ward and nursing home levels).

## Methods

This study used data from the Danish Observational Study of Eldercare work and musculoskeletal disorderS (DOSES)(Karstad *et al.*, 2018) – a Danish cohort of workers in eldercare collected from September 2013 to December 2014. Ethical approval for DOSES was provided by the Danish Data Protection Agency and the Ethics Committee for the regional capital of Denmark (H-4-2013-028). Written, informed consent was obtained from all participants. The full details of DOSES have been previously published (Karstad *et al.*, 2018). As such, we will only describe aspects specifically relevant to this study below. Throughout this manuscript we use the term determinants to describe the factors we collect that may potentially influence quantitative demands. We emphasize that we do not use the term ‘determinants’ in a causal sense. Rather, we use ‘determinants’ in the statistical sense, i.e. factors that can explain the variance in another variable, irrespective of whether they are causal factors.

### Participants

We purposively selected and invited 83 nursing homes located in Zealand and the capital region of Denmark to participate in the study. The aim of the strategic selection was to include nursing homes of various sizes and care models. Of the 83 nursing homes invited, 20 nursing homes agreed to participate and were subsequently included. After a nursing home agreed to participate, we distributed written information about the aim and activities of the research to all employees and arranged an information meeting at the nursing home to inform employees about the study and invite them to participate. Participants in the study were eldercare workers from 18–65 years of age, employed in nursing homes more than 15 h/week on day and evening shifts and spending a minimum of 25% of their working time on tasks related to direct care of residents.

### Data collection

Only baseline data were used for this multi-level cross-sectional study. Baseline data collection for workers included a structured self-administered questionnaire, a health check which recorded technical measures of health and physical capacity, and an observation period where workers were directly observed going about their tasks and work schedules, and physical activity at work and leisure was assessed using accelerometers. Baseline data collection for nursing home managers and team managers (responsible for the wards) consisted of a web-based questionnaire about formal and informal organizational structures at the nursing home and wards.

### Primary outcome

The primary outcome of this study was self-reported quantitative demands, collected at baseline. To measure quantitative demands, we used two items from the Copenhagen Psychosocial Questionnaire (COPSOQ) II (Pejtersen *et al.*, 2010). These were: ‘Do you get behind with your work?’ and ‘Do you have enough time for your work tasks?’. Workers responded on a 5-point Likert scale with possible response values of ‘always’, ‘often’, ‘sometimes’, ‘rarely’ or ‘never/almost never’ The responses to these two items were then averaged and converted to a 0–100 scale with higher values indicating higher quantitative demands.

### Determinants at the worker level

Most worker level determinants were collected with the baseline questionnaire. This included age (years), sex (male/female), country of birth (Denmark/outside Denmark), smoking habits (daily/occasional/former/never) and the proportion of time spent conducting: direct care tasks, support tasks, administration tasks (5-point Likert scale: rarely/never, roughly 1/4 of the time, roughly 1/2 of the time, roughly 3/4 of the time, almost all the time). Workers’ job was divided into three categories, i.e. ‘care helpers’ (who had 14 months of training in care provision), ‘care aides’ (who had completed an additional 6 months of training) and ‘nurses or other health professionals’. We defined seniority as the number of years worked in the current position. Workers were classified according to the type of shift they normally worked: ‘fixed day’, ‘fixed evening’, ‘day/evening’ or ‘other’.

Information about the psychosocial work environment was collected using questions from the COPSOQ II (Pejtersen *et al.*, 2010). The psychosocial aspects of work collected were: influence at work (2 items: ‘Do you have a large degree of influence concerning your work?’, ‘Can you influence the amount of work assigned to you?’), social support from colleagues (2 items; ‘How often are your colleagues willing to listen to your problems at work?’, ‘How often do your colleagues talk with you about how well you carry out your work?’), quality of leadership (4 items; ‘To what extent would you say that your immediate superior: -makes sure that the individual member of staff has good development opportunities?’, ‘-gives high priority to job satisfaction?’, ‘-is good at work planning?’, ‘-is good at solving conflicts?’) and emotional demands (4 items; ‘Does your work put you in emotionally disturbing situations?’, ‘Do you have to relate to other people’s personal problems as part of your work?’, ‘Is your work emotionally demanding?’, ‘Do you get emotionally involved in your work?’). Each item was collected on a 5-point Likert scale. For use in the analyses, all items within each psychosocial aspect were averaged and converted to a 0–100 scale. Further, height and weight were measured by trained clinical personnel to calculate BMI (kg/m²).

We collected step-rate (steps/hour) at work using accelerometry. Participants were asked to wear three accelerometers (on the thigh, upper back and dominant arm) for a minimum of four consecutive days, including at least two working days. Participants allergic to patches were excluded from wearing the accelerometers. The accelerometers used were ActiGraph GT3X+ accelerometers (ActiGraph, Florida, United States). A validated software program (Acti4)(Skotte *et al.*, 2014) was applied for analysing the accelerometer data and counting steps with very high sensitivity and specificity (Ingebrigtsen *et al.*, 2013). Participants were also asked to keep diary recording the time when they started and finished work. We then used these diaries to classify the steps recorded into occurring either in, or outside work. To be considered a valid representation of steps taken, accelerometers needed to collect data for at least 4 h or 75% of that shift. The step-rate was then calculated by dividing the total number of steps taken at work during the measurement days by the total number of hours worked, including only those steps/time periods that were deemed valid.

As direct care tasks are the core work of eldercare, we conducted direct observations of the care work performed by each worker to record their physical and psychosocial work exposures. These observations were conducted at baseline by trained observers following a strict protocol that was developed based on previous observational studies in the workplace (Karstad *et al.*, 2018) using tablets and the Noldus Observer XT pocket observer program (Noldus, Wageningen, The Netherlands). Since over 70% of all handlings occur during a period of 4 h in the morning and 4–5 h in the evening, observations were limited to these two time periods. As such, we recorded, to the extent possible, all handlings occurring in each ward over a single day in a period of 4 h during day shifts and 4–5 h during evening shifts. From these observations, exposures to direct care tasks for each eldercare worker were calculated as an exposure per shift by summing up the average exposures for each resident under their care. The exposures recorded during the observations were the number of: resident handlings (lifting, repositioning and turning of the resident; total), resident handlings without support from the resident, resident handlings without support from colleagues, squats performed, push/pull tasks (e.g. of a wheelchair), disturbances/interruptions and obstacles (e.g. a needed lifting aid was not available).

### Determinants at the ward and nursing home level

All determinants at the ward and nursing home level were obtained from ward and nursing home managers using the baseline questionnaire. The wards were divided into four categories – somatic, dementia, temporary rehabilitation, and psychiatric. The ward/home size was defined as the maximum number of residents that could be allocated to that ward/home. We obtained information about the usual resident/staff ratio on day and evening shifts by asking “what were the usual number of residents on the ward?” and “how many staff are usually working on day [and evening] shifts?” The resident staff ratio was then calculated as the number of residents divided by the number of staff for each shift (day/evening). Workers only working on day or evening shifts received the respective ratio for their shift, Workers working both day and evening shifts were allocated an average of the ratio for day and evening shifts. We asked about the availability of rooms for taking breaks (yes/no), whether it was permissible to take breaks from work (yes/no), the number of floors (n) and the presence of elevators (yes/no). We also obtained information about the location of various aid devices used by the elder care workers. This was done via a combined score that incorporated the responses to questions that asked where these devices were located (4 response categories: in the room; in the corridor; in the ward; outside the ward) for the eight most commonly used aid devices (floor lift, stand-up lift, ceiling lift, sail, easy slide/slide sheet, glide board, transfer belt, support sock). If a device was not available, it did not contribute towards the score developed. Information about the underlying principles for allocation of residents to workers across a ward were asked with a series of questions ‘To what extent is the allocation of work tasks distributed: -fairly?’, ‘-according to worker health?’, ‘-according to job title’, ‘-so that workers have an equal balance of physical work?’, ‘-to maintain workers with the same residents?’ and ‘-according to residents’ needs?’. Responses to these questions were collected on a 5-point Likert scale (‘to a very large extent’, ‘to a large extent’, ‘somewhat’, ‘to a small extent’, ‘to a very small extent’). Perceived general health was obtained from workers using a single item from the SF-36 (Brazier *et al.*, 1992) ‘In general, would you say your health is:’ with 5 possible responses – ‘excellent’, ‘very good’, ‘good’, ‘fair’ or ‘poor’.

### Statistical analysis

This exploratory analysis had two main parts. The first part was the investigation of the proportion of variance in quantitative demands occurring at each of the three hierarchical levels i.e. between workers (within wards), between wards (within nursing homes) and between nursing homes. This was conducted using Variance Components Analysis (VCA). VCA is a particular form of mixed-effects modelling that includes only random effects. As such, we constructed a mixed-effects linear regression model that included only the factors worker, ward and nursing home as random intercepts.

The second part of the analysis was the investigation of potential determinants of quantitative demands at each of the three levels. We did this in two steps. First, we conducted a univariate analysis that individually assessed each candidate determinant as a fixed-effect in the random-intercept model described above. The second step then combined all significant determinants (*P* < 0.05) into a single multivariate model. We present β-coefficients with confidence intervals, and marginal R^2^ values for all models investigated. Because of the multiple tests conducted, Holm-Šidàk adjusted p-values (Ludbrook, 1998) are provided alongside the standard *P*-values for all univariate models.

All statistical analyses were conducted in R (R Core Team, 2018)/ RStudio (RStudio Team, 2016) using packages: lme4 (Bates *et al.*, 2019); VCA (Andre and Dufey, 2020); broom.mixed (Bolker *et al.*, 2020); DHARMa (Hartig, 2020); insight (Lüdecke *et al.*, 2020); and the tidyverse suite of packages (Wickham *et al.*, 2019). An α of 0.05 was used for all significance testing.

We conducted two sensitivity analyses in this study. Firstly, because of a high correlation between the type of shift (having a fixed evening shift) and staffing ratio (r = −0.82), we conducted an extra analysis for staffing-ratio that was stratified by the type of shift. This analysis was limited to two strata (Fixed day shift and Fixed evening shift) due to the low number of workers outside these two categories. Secondly, due to concerns regarding common method variance between our psychosocial measures (influence at work, social support from colleagues, quality of leadership and emotional demands) and quantitative demands, we conducted a sensitivity analysis that used ward-level measures of these variables. To calculate the ward-level measures of these variables we averaged the response of all workers in that ward.

## Results

Of the 553 workers included in DOSES, 383 provided the relevant data to be included in this study (Table 1). These 383 workers were generally middle aged (mean [SD] = 45.4 [10.5]), born in Denmark (80.2%) and almost all were female (96.1%). Most (75.5%) worked in somatic wards and almost all were employed as either care aides (48.7%) or care helpers (42.6%). Most (85.1%) rated their health as ‘good’ or better.

**Table 1.** Demographic characteristics of workers in eldercare.

Demographics (*n* = 383)	Mean (SD)	*n* (%)
Age (years)	45.4 (10.5)	-
Sex (female)	-	368 (96.1%)
Country of birth (Denmark vs other)(*n* = 369)		296 (80.2%)
BMI (kg/m^2^) (*n* = 379)	26.1 (5.1)	-
Smoking habits		
Never smoked	-	126 (32.9%)
Former smoker	-	116 (30.3%)
Occasional smoker	-	25 (6.5%)
Daily smoker	-	116 (30.3%)
Perceived health (*n* = 378)		
Excellent	-	10 (2.6%)
Very good	-	104 (27.5%)
Good	-	208 (55.0%)
Not so good	-	53 (14.0%)
Poor	-	3 (0.8%)
Job position (*n* = 380)		
Care aide	-	185 (48.7%)
Care helper	-	162 (42.6%)
Nurse or other health professional	-	33 (8.7%)
Type of ward		
Somatic	-	289 (75.5%)
Dementia	-	77 (20.1%)
Temporary rehabilitation	-	9 (2.3%)
Psychiatric	-	8 (2.1%)
Perceived quantitative demands	44.7 (20.3)	

### Proportion of variance in quantitative demands occurring at each level

In our sample of eldercare workers, the mean (SD) level of quantitative demands was 44.7 (20.3). A histogram and cumulative frequency curve are provided in the online Appendix – Fig. S1(available at *Annals of Occupational Hygiene* online). The variance in quantitative demands occurred primarily between workers within wards (90.9%) while the variance in quantitative demands that occurred at the ward and nursing home levels was 1.1% and 8.1% respectively.

### Potential determinants of quantitative demands at each level

In the univariate models (Table 2), significant determinants of quantitative demands (0–100 scale) occurred at the worker and ward levels. At the worker level, decreased quantitative demands was associated with being a care helper (compared to a care aide; β = −10.4 [−14.5; −6.1]), being born outside Denmark (β = −9.3 [−14.3; −4.2]), working on fixed evening shifts (β = −11.5 [−16.5; −6.6]), increased influence at work (0–100 scale; β = −0.2 [−0.3; −0.1]), increased quality of leadership (0–100 scale; β = −0.3 [−0.4; −0.2]) and decreased emotional demands (0–100 scale; β = 0.4 [0.2; 0.5]). At the ward level, an increased resident/staff ratio was associated with a decrease in quantitative demands (0–100 scale; β = −1.5 [−2.6; −0.3]), however using the Holm-Šidàk adjusted *P*-value this association was no longer significant. The marginal R^2^ values (R^2^ values for the fixed effects only) for each determinant ranged from <0.01 to 0.08 with the highest value being for emotional demands. Further details for all potential determinants tested are presented in Table 2. Furthermore, descriptive statistics for all potential determinants are provided in the online Appendix – Table S1 (available at *Annals of Occupational Hygiene* online).

**Table 2.** Univariate analyses of all determinants on quantitative demands in eldercare.

Determinant	R^2^_m_	Estimate [±95%CI]	*P*-value	
			Standard	Holm adj.
Individual level variables				
Age	<0.01	−0.1 [−0.3; 0.1]	0.147	
Sex (Female)	<0.01	5.7 [−4.6; 15.9]	0.277	
BMI	<0.01	−0.4 [−0.7; 0.0]	0.078	
Job position (ref: Care Aide)	0.06			
Care helper		**−10.4 [−14.5; −6.1]**	**<0.001**	**<0.001**
Nurse or other health professional		−1.3 [−8.5; 6.0]	0.727	
Seniority	<0.01	0.0 [0.0; 0.0]	0.084	
Smoking habits	<0.01	1.7 [−2.1; 5.6]	0.380	
Birth (outside Denmark)	0.03	**−9.3 [−14.3; −4.2]**	**<0.001**	**0.013**
Shifts worked (ref: Fixed Day)	0.05			
Fixed Evening		**−11.5 [−16.5; −6.6]**	**<0.001**	**<0.001**
Day/Evening		−1.7 [−8.6; 5.5]	0.630	
Other		−4.5 [−13.5; 5.3]	0.319	
Influence at work	0.05	**−0.2 [−0.3; −0.1]**	**<0.001**	**<0.001**
Social support from colleagues	<0.01	−0.1 [−0.2; 0.0]	0.081	
Quality of leadership	0.06	**−0.3 [−0.4; −0.2]**	**<0.001**	**<0.001**
Emotional demands	0.08	**0.4 [0.2; 0.5]**	**<0.001**	**<0.001**
Proportion of time spent in:				
Care work	0.01	−2.3 [−8.1; 3.6]	0.429	
Support work (cleaning etc)	<0.01	−2.8 [−10.6; 5.0]	0.478	
Administration work	0.03	−6.8 [−15.7; 2.0]	0.131	
Step/hour at work	<0.01	−0.006 [−0.013; 0.001]	0.107	
Number of resident handling tasks (lifts/repositions/turnings):				
Total	<0.01	0.0 [0.0; 0.1]	0.220	
Without patient support	<0.01	0.0 [0.0; 0.1]	0.164	
Without help from colleagues	<0.01	0.0 [0.0; 0.1]	0.420	
Number of squats	<0.01	0.0 [0.0; 0.0]	0.845	
Number of disturbances/interruptions	<0.01	0.0 [−0.1;0.1]	0.610	
Number of obstacles	<0.01	0.1 [0.0;0.2]	0.077	
Number of push/pull tasks (e.g of a wheelchair)	<0.01	0.0 [0.0; 0.1]	0.213	
**Ward level variables**				
Ward Type (ref: somatic)	<0.01			
Dementia		1.7 [−4.0; 7.2]	0.552	
Temporary rehab		3.1 [−11.3; 17.4]	0.676	
Independent living		3.2 [−13.1; 19.3]	0.697	
Ward size (max n residents)	<0.01	−0.2 [−0.8; 0.3]	0.415	
Staffing-ratio (residents/staff)	0.02	**−1.5 [−2.6; −0.3]**	**0.009**	0.315
Rooms for breaks (yes)	<0.01	0.3 [−8.5; 9.8]	0.948	
Permission for breaks (yes)	0.01	−5.4 [−11.8; 1.0]	0.106	
Location of aides	<0.01	7.2 [−3.7; 18.3]	0.207	
To what extent is the allocation of work tasks (residents):				
Distributed fairly	0.01	−4.6 [−14.0; 4.6]	0.337	
According to worker health	0.02	2.8 [−8.8; 14.3]	0.642	
According to job title	0.01	−4.3 [−11.0; 2.6]	0.205	
So that workers have the right balance of physical work	0.02	−3.4 [−17.5; 10.6]	0.643	
To maintain workers with the same residents	<0.01	3.3 [−9.0; 15.5]	0.615	
According to residents’ needs	0.01	7.0 [−4.0; 18.4]	0.234	
**Home level variables**				
Home size (max n residents)	<0.01	0.0 [−0.2;0.1]	0.614	
Number of floors	<0.01	−1.0 [−4.5; 2.5]	0.593	
Presence of elevators (No)	<0.01	4.5 [−5.5; 14.6]	0.394	

R^2^_m_ – marginal R^2^: proportion of total variance explained by the fixed effects in the model only.

Bold values indicate an association was statistically significant at the 0.05 level.

When combined in the multivariate analysis two determinants markedly changed their effect estimates (Table 3). This suggests they share a causal chain with other factors assessed in the model. These were, the effect of evening shift (and evening/night shift) which became stronger when included in the full multivariate model (change (Δ) β = 5.1 and 11.4 respectively), and the resident/staff ratio, which reversed its direction of effect (Δβ = 3), now showing a positive association with quantitative demands. Determinants that were associated with a significant reduction in quantitative demands in the multivariate model (Table 3) were being born outside Denmark (β = −6.5 [−11.0; −2.0]), having a job as a care helper (β = −8.1 [−11.9; −4.1]), working fixed evening and evening/night shifts (compared to day shifts; β = −16.6 [−24.7; −8.6] and −22.7 [−41.4; −3.7] respectively) and influence at work (β = −0.2 [−0.3; −0.1]). Increased emotional demands was associated with an increase in quantitative demands (β = 0.3 [0.2; 0.4]). The marginal R^2^ and conditional R^2^ values for the multivariate model were 0.24 and 0.34 respectively.

**Table 3.** Multivariate analysis of selected determinants on quantitative demands in eldercare.

Determinant	Estimate [±95%CI]	*P*-value
Birth (outside Denmark)	**−6.5 [−11.0; −2.0]**	**0.006**
Work Job (ref: Care Aide)		
Care Helper	**−8.1 [−11.9; −4.1]**	**<0.001**
Nurse or other health professional	0.3 [−6.2; 6.9]	0.931
Work Shift (ref: Day shift)		
Fixed Evening	**−16.6 [−24.7; −8.6]**	**<0.001**
Day/Evening	−4.1 [−10.9; 2.9]	0.253
Evening/Night	**−22.7 [−41.4; −3.7]**	**0.021**
Day/Night	0.8 [−32.4; 34.3]	0.962
Day/Evening/Night	−8.1 [−17.7; 1.8]	0.099
Other	14.1 [−19.5; 47.0]	0.411
Quality of leadership	−0.1 [−0.2; 0.0]	0.081
Emotional demands	**0.3 [0.2; 0.4]**	**<0.001**
Influence at work	**−0.2 [−0.3; −0.1]**	**<0.001**
Staffing-ratio (residents/staff)	1.5 [−0.4; 3.4]	0.128

R^2^_m_ = 0.24; R^2^_c_ = 0.34

R^2^_m_ – marginal R^2^: proportion of total variance explained by the fixed effects in the model only. R^2^_c_ – conditional R^2^: proportion of total variance explained by both fixed and random effects in the model.

### Sensitivity analyses

When stratifying by the type of shift that workers conducted (fixed day or fixed evening; univariate model), an increased resident/staff ratio was associated with increased quantitative demands. This association was stronger in day workers (β = 5.7 [0.8; 10.9]; *P* = 0.026; R^2^_m_ = 0.03) than in evening workers (β = 1.6 [−0.7; 3.9]; *P* = 0.170; R^2^_m_ = 0.03). When using ward-level aggregates of our potential psychosocial determinants (influence at work, social support from colleagues, quality of leadership and emotional demands) results were similar to our main results (Online Appendix – Table S2, available at *Annals of Occupational Hygiene* online). The effect size and R^2^_m_ increased slightly for influence at work, but decreased slightly for the other three variables. There were no changes in statistical significance.

## Discussion

### Summary of findings

In this study, we identified that nearly all of the variability in the perceived quantitative demands at work occurred between workers (within wards), and that only a minor part of the variability occurred at higher organisational levels in eldercare workplaces, i.e. wards (within nursing homes) and between nursing homes. Furthermore, we found that factors in the psychosocial work environment (i.e. emotional demands, quality of leadership and influence at work) were the strongest determinants for perceived quantitative demands in eldercare. Organisational determinants also determining perceived quantitative demands were the job position, type of shift, and resident/staff ratio.

### Strengths and limitations

The primary strengths of this paper are the collection of data at multiple organisational levels from 20 nursing homes, and the workplace observational methodologies that were used to collect much of the data. The primary weakness of this paper is its cross-sectional study design, which increases the risk for common-method variance and means that we cannot draw conclusions, neither regarding causality nor reverse causality. For instance, some data (particularly regarding the psychosocial work environment) were based on self-report by the eldercare workers, which might influence the associations with the reported perceived quantitative demands at work. However, we conducted sensitivity analyses using ward-aggregated versions of these variables and had similar results. We also do not assess any potential moderating effects among the tested determinants. The multiple univariate tests conducted in this analysis also makes it more likely to find significant effects which do not truly exist (i.e. type II errors). However, this concern is minimised through the use of Holm-Šidàk adjusted *P*-values (i.e. *P*-values adjusted based upon the number of tests conducted) and inclusion of a multivariate model. Finally, we were unable to assess many potential determinants that may be of importance for understanding quantitative demands. For example, administration demands, number of vacant positions (e.g. due to sickness absence) or number of temporary employees hired to fill those vacant positions.

### Comparisons with other studies

In contrast to our own, most studies on perceived quantitative demands have focussed on the consequences of high perceived quantitative work demands (Hansen *et al.*, 2012; Clausen *et al.*, 2014a), with very few studies having investigated the determinants of the quantitative demands, nor tried to understand determinants of quantitative demands at multiple organisational and individual levels together in the same analysis. However, despite their scarcity, these studies do seem to agree with our own. Two Danish studies (one in eldercare) showed that evening shifts were associated with reduced quantitative demands compared to day shifts (Bøggild *et al.*, 2001; Nabe-Nielsen *et al.*, 2009). Other studies support our finding of a relationship between psychosocial aspects of the work environment and perceived quantitative demands (Rugulies *et al.*, 2010; Hansen *et al.*, 2015; Elfering *et al.*, 2017; Mette *et al.*, 2018; Burr *et al.*, 2019; Berthelsen *et al.*, 2020; Van Den Oetelaar *et al.*, 2021). For example, one study in hospital nurses showed that increased social support from colleagues was associated with a decrease in quantitative demands (Van Den Oetelaar *et al.*, 2021). Another example is a controlled trial in eldercare that found that self-rostering (i.e. increasing workers’ influence over their tasks) decreased self-reported quantitative demands (Hansen *et al.*, 2015).

Many studies have investigated the influence of occupation on quantitative demands (e.g. comparing manufacturing and eldercare), but such investigations are not useful for us in our aim to understand the determinants of quantitative demands within eldercare. Furthermore, although there are many ways to take occupational variation into account (Bültmann *et al.*, 2002; Madsen *et al.*, 2018; Niedhammer *et al.*, 2018), we used multivariate analysis to understand the impact of our proposed determinants while holding other potential determinants still. This was done because we wanted to maintain a purely hierarchical model, which prohibited the inclusion of occupation in the multi-level modelling since different occupations (e.g. care helpers, care aides and nurses) are spread over the different wards.

### Interpretations and implications

Overall, we observed that nearly all of the variability in perceived quantitative demands at work occurred between workers within wards, and that only a minor part of the variability is explained at higher organisational workplace levels in eldercare. In other words, different workers within a ward likely have different exposures/tasks/demands. This may indicate that workplace interventions should mainly focus on the worker level when seeking to modify quantitative demands. However, since most of the determinants identified are organisational determinants, it seems more likely that unidentified moderating factors are causing upper-level determinants to cause worker-level variation. Since many of the organisational determinants we found were non-modifiable (i.e. job position, type of shift), we must consider what it is about these non-modifiable determinants that is related to perceived quantitative demands. For example, the resident-staff ratio was strongly related to the type of shift, which confounded its relationship with perceived quantitative demands. As such it may be that interventions that reduce the resident-staff ratio may, despite the mixed results found in our study, bring about significant reductions in perceived quantitative demands.

Another important result was that we found psychosocial work environment factors to be the strongest determinants of perceived quantitative demands in eldercare. This contrasts strongly with the physical work tasks assessed, such as resident handlings and push/pull tasks, which were not associated with perceived quantitative demands. As such, it seems that perceived quantitative demands measure psychosocial aspects of work, rather than physical exposures such as resident handlings. For instance, it may be that physical work that occurs within a positive environment (e.g. one where the worker has influence on their work and emotional demands are minimal) do not factor as quantitative demands in the minds of workers. It may also be that the ‘time pressure’ aspect of perceived quantitative demands as measured in the present study takes supremacy and psychosocial factors are more closely associated with not having enough time to accomplish your work than any particular physical factor.

### Future research

To better-inform research-based preventive interventions for reducing high perceived quantitative demands, we encourage more studies which investigate the determinants of perceived quantitative demands, both to identify determinants that were not included in our study and to replicate our findings. It is also important that we engage practitioners, researchers in health and researchers dealing with work organization and management in order to gain a full picture of these determinants (MacDonald *et al.*, 2008), collect them from multiple levels of the workplace and use methods less prone to bias (i.e. technical measures or observation) where possible. Developing and testing interventions targeted at identified determinants to reduce perceived quantitative demands is, alongside appropriate process monitoring, the next step in reducing quantitative demands among eldercare workers (Van Der Beek *et al.*, 2017).

## Conclusion

We found that the variation in perceived quantitative demands of eldercare workers was mainly explained by individual differences (within wards), and only to a small extent by differences between wards (within nursing homes) and between nursing homes. Our study indicates that psychosocial work environment factors (i.e. emotional demands, influence at work, and quality of leadership) are the strongest determinants for perceived quantitative demands in eldercare. In contrast, physical work tasks (e.g. number of resident handling tasks, push/pull tasks) seem only marginally related to perceived quantitative demands. Organisational determinants such as the job position, type of shift, and resident-staff ratio are also important.

## Supplementary Material

wxac039_suppl_Supplementary_Material

## Data Availability

The data can be made available upon reasonable request.
