# Supplementary material for: Nursing Home, Ward and Worker Level Determinants of Perceived Quantitative Work Demands: A Multi-Level Cross-Sectional Analysis in Eldercare
Source: Ann Work Expo Health. 2022 Jun 23;66(8):1033–43. doi: 10.1093/annweh/wxac039 (PMC9551322; doi:10.1093/annweh/wxac039)
Supplement: wxac039_suppl_Supplementary_Material [file wxac039_suppl_supplementary_material.pdf]

Nursing home, ward and worker level determinants of perceived quantitative work demands: a multi-level cross-sectional analysis in eldercare

Online Appendix

Matthew L Stevens, Kristina Karstad, Leticia Bergamin Januario, Svend Erik Mathiassen, Reiner Rugulies, David M Hallman, Andreas Holtermann

Figure S1: Histogram and Cumulative Frequency Curve for Perceived Quantitative Demands of Eldercare Workers

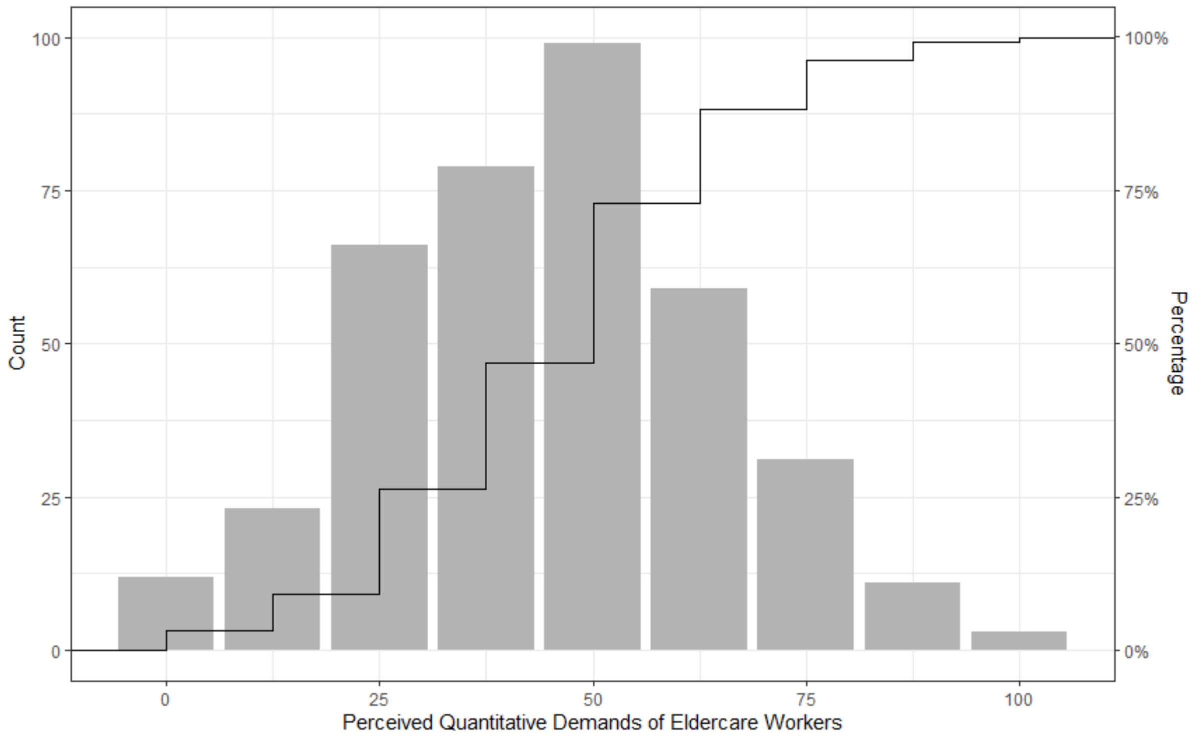

Table S1: Descriptives for all investigated potential determinants of perceived quantitative demands among Danish eldercare workers

**Potential Determinant**	**Mean (SD), Median (IQR) or n (%)**
**Individual level variables (n=383)**	
Age (years)	45.4 (SD 10.5)
Sex (female)	368 (96.1%)
BMI (kg/m2) (n=379)	26.1 (SD 5.1)

Job Position	
Care Aide	185 (48.7%)
Care Helper	162 (42.6%)
Nurse or other health professional	33 (8.7%)
Seniority (time in current position; years)	17 (SD 10.8)
Smoking	
Never smoked	126 (32.9%)
Former smoker	116 (30.3%)
Occasional smoker	25 (6.5%)
Daily smoker	116 (30.3%)
Country of Birth (Denmark vs other) (n=369)	296 (80.2%)
Shifts Worked	
Fixed Day	219 (57.2%)
Fixed Evening	81 (21.1%)
Day/Evening	50 (13.1%)
Other	33 (8.6%)
Influence at work	57.1 (SD 19.0)
Social support	67.8 (SD 16.8)
Quality of leadership (n=380)	61.2 (SD 16.8)
Emotional demands (n=382)	51.5 (SD 15.3)
Proportion of time spent in:	
Care work (n=373)	
Rarely/never	0 (0.0%)
Approx ¼ of the time	27 (7.2%)
Approx ½ of the time	115 (30.8%)
Approx ¾ of the time	112 (30.0%)
Almost all the time	119 (31.9%)
Support work (cleaning etc; n=343)	
Rarely/never	44 (12.8%)
Approx ¼ of the time	206 (60.1%)
Approx ½ of the time	59 (17.2%)
Approx ¾ of the time	29 (8.5%)
Almost all the time	15 (4.4%)
Administration work (n=342)	
Rarely/never	11 (3.2%)
Approx ¼ of the time	29 (8.5%)
Approx ½ of the time	52 (15.2%)
Approx ¾ of the time	230 (67.3%)
Almost all the time	30 (8.8%)
Step/hour at work	1114.4 (SD 274.7)
Number of resident handling tasks (lifts/repositions/turnings):	

Total	56.0 (IQR 26.1 to 94.0)
Without patient support	40.7 (IQR 18.0 to 77.0)
Without help from colleagues	28.0 (IQR 12.0 to 52.5)
Number of squats	37.0 (IQR 14.3 to 78.0)
Number of disturbances/interruptions	16.0 (IQR 7.3 to 33.0)
Number of obstacles	12.0 (IQR 5.5 to 27.0)
Number of push/pull tasks (e.g of a wheelchair)	24.5 (IQR 11.0 to 47.0)
**Ward level variables (n=95)**	
Ward Type	
Somatic	72 (75.8%)
Dementia	19 (20.0%)
Temporary rehabilitation	2 (2.1%)
Psychiatric	2 (2.1%)
Ward size (max n residents)	11 (IQR 9 to 14)
Staffing-ratio (residents/staff)	3.7 (IQR 3.0 to 5.0)
Rooms for breaks (yes)	88 (92.6%)
Permission for breaks (yes)	73 (76.8%)
Location of aides (n=90)	1.5 (0.3)
To what extent is the allocation of work tasks (residents):	
Distributed fairly	
To a very small extent	0 (0.0%)
To a small extent	4 (4.2%)
Somewhat	20 (21.1%)
To a large extent	61 (64.2%)
To a very large extent	10 (10.5%)
According to worker health	
To a very small extent	0 (0.0%)
To a small extent	1 (1.1%)
Somewhat	19 (20.0%)
To a large extent	52 (54.7%)
To a very large extent	23 (24.2%)
According to job title	
To a very small extent	0 (0.0%)
To a small extent	6 (6.3%)
Somewhat	21 (22.1%)
To a large extent	51 (53.7%)
To a very large extent	17 (17.9%)
So that workers have the right balance of physical work	
To a very small extent	0 (0.0%)

To a small extent	1 (1.1%)
Somewhat	50 (52.6%)
To a large extent	38 (40.0%)
To a very large extent	6 (6.3%)
To maintain workers with the same residents	
To a very small extent	1 (1.1%)
To a small extent	15 (15.8%)
Somewhat	33 (34.7%)
To a large extent	38 (40.0%)
To a very large extent	8 (8.4%)
According to residents' needs (n=94)	
To a very small extent	5 (5.3%)
To a small extent	28 (29.8%)
Somewhat	39 (41.5%)
To a large extent	20 (21.3%)
To a very large extent	2 (2.1%)
**Home level variables (n=20)**	
Home size (max n residents) (n=17)	78.6 (SD 28.9)
Number of floors	2 (IQR 1 to 3)
Presence of elevators (Yes)	17 (85.0%)

Table S2: Sensitivity Analyses - ward-level aggregates of potential psychosocial determinates on quantitative demands in eldercare workers

Determinant	R^2_m	Estimate [±95%CI]	p-value
**Ward level variables**			
Influence at work	0.06	**-0.4 [-0.7; -0.2]**	**<0.001**
Social support	<0.01	-0.1 [-0.4; 0.1]	0.416
Quality of leadership	0.03	**-0.3 [-0.5; -0.1]**	**0.006**
Emotional demands	0.02	**0.3 [0.1; 0.6]**	**0.012**
R^2_m – marginal R^2; proportion of total variance explained by the fixed effects in the model only			
